# Multi-Pathway Mechanisms of Beef in Ameliorating Spleen Deficiency Syndrome: Insights from Digestive Function, Immunity, and Gut Microbiota

**DOI:** 10.3390/foods15030488

**Published:** 2026-02-01

**Authors:** Ying Zhang, Ang Ru, Xinghui Wang, Ke Wang, Xueyuan Bai, Xinjun Zhang, Chaozhi Zhu, Gaiming Zhao

**Affiliations:** 1 Henan Key Lab of Meat Processing and Quality Safety Control, Henan Agricultural University, Zhengzhou 450002, China; 2 College of Food Science and Technology, Henan Agricultural University, Zhengzhou 450002, China; 3 Ningxia Xiahua Meat Food Co., Ltd., Zhongwei 755000, China

**Keywords:** medicine food homology, tonifying spleen, beef, Chinese yam, healthy foods

## Abstract

Beef and yam are valued as functional foods, yet their synergistic effects on gastrointestinal health and immunity remain underexplored. This study investigated the effects of beef and yam on the spleen and stomach. In the present study, a rat model of spleen deficiency was established by poor diet and exhaustive swimming. The weight, food intake, gastrointestinal and immune indexes, and the intestinal flora of the rats were examined. The results showed that the levels of gastrin, motilin, and four cytokines improved. Specifically, the beef group exhibited marked recovery in gastrointestinal hormones, with serum gastrin and motilin levels increasing to approximately 60 pg/mL and 70 pg/mL, respectively, close to the normal control levels, and significantly higher than the model group. The beef and yam effectively restored the balance of intestinal flora, which significantly increased the diversity of intestinal microorganisms. In addition, the tissue structure of the spleen, stomach, small intestine, and colon was also effectively improved. Additionally, yam increased gut microbial diversity and optimized the microbial community structure, consequently enhancing the overall health status. This study elucidates the multi-pathway mechanisms by which beef and yam ameliorate spleen deficiency, providing a scientific basis for their application in functional foods.

## 1. Introduction

Spleen deficiency syndrome (SDS) is an impairment of the spleen’s function of transportation and digestion, leading to multi-system and multi-organ dysfunction, especially digestion and immunity [1]. Meanwhile, research indicated that the occurrence and development of spleen deficiency were closely related to intestinal inflammatory lesions and water metabolism disorders [2]. D-xylose is a pentose sugar absorbed by the small intestine after oral administration. Under normal circumstances, D-xylose is virtually absent in the bloodstream. Therefore, detecting D-xylose levels in the blood following ingestion of a specific dose of D-xylose solution allows for indirect assessment of the intestinal mucosa’s absorption function [3]. Thus, the digestion–absorption function of rats was reflected by gastrointestinal transit rate and D-xylose levels in serum [4]. SDS has grown into an increasingly non-negligible health issue. Although it does not directly threaten life, it significantly impairs overall health and quality of life. Consequently, research on SDS has garnered escalating attention from experts and scholars in recent years. Currently, most research related to the treatment of SDS mainly focuses on regulating gastrointestinal and immunomodulatory functions [5,6,7].

The medicine and food homology concept has occupied an important position in traditional Chinese culture since ancient times, which mainly emphasizes the close connection and mutual transformation between food and medicine [8]. The concept of food as medicine echoes the famous maxim of Hippocrates, the pioneer of modern medicine: “Let food be your medicine, and medicine be your food” [9]. In both traditional health practices and contemporary nutritional science, the maintenance of gastrointestinal integrity and immune competence is recognized as a cornerstone of systemic well-being. Dietary components such as beef and Chinese yam (Dioscorea) have been historically utilized across cultures to support digestive function. Beef is a nutritious food rich in high-quality proteins and amino acids, which is an important source of energy [10]. Research indicated that beef is rich in all protein amino acids and physiologically essential non-protein amino acids (taurine and β-alanine). Therefore, beef is recognized as a functional food that optimizes human growth, development, and health [11,12]. Chinese yam contains notable levels of mucilaginous polysaccharides, resistant starch, minerals, and antioxidant compounds, which have demonstrated potential in modulating gut barrier function and metabolic homeostasis in preliminary studies [13]. By contrast, the law of purgation of the spleen and stomach is often accompanied by pungent dispersing herbs, such as dry ginger “warming the middle and dispersing the cold” [14].

Despite this established nutritional and phytochemical basis, a significant mechanistic gap persists in our understanding. Current research has largely been siloed, focusing on isolated nutrients or singular physiological outcomes. For instance, studies on beef often emphasize its role in muscle protein synthesis or specific nutrient bioavailability, while investigations into Chinese yam frequently center on its effects on blood glucose or lipid metabolism. A critical, yet underexplored, area is the integrative and synergistic impact of these whole foods on the complex gut–immune axis. This gap is particularly relevant in the context of spleen deficiency syndrome (SDS), a condition associated with both digestive and immune dysfunction. A systems-level investigation that examines the concurrent modulation of this axis could provide transformative insights into their holistic mechanism of action.

To bridge this knowledge gap, the present study was designed to evaluate the hypothesis that beef, Chinese yam, and dried ginger exert restorative effects in SDS through coordinated modulation of gastrointestinal and immune functions, potentially mediated by the gut microbiota. This study used a dual-factor composite method combining poor diet and exhaustive swimming to construct the SDS model. The effects of beef, Chinese yam, and dried ginger in SDS were evaluated by analyzing immune indicators, gastrointestinal digestive indicators, and tissue pathology sections. Also, 16S rRNA sequencing was performed on rat feces to detect changes in the intestinal microbiota and reveal its mechanism of action. The findings are intended to provide a robust, evidence-based scientific rationale for the strategic use of these common foods in functional dietary formulations aimed at promoting gastrointestinal resilience and immune homeostasis.

## 2. Materials and Methods

### 2.1. Materials and Chemicals

The raw material used was imported bovine scapular region meat (18 months) from Brazil (Matupa, Brazil). The Chinese yam was the authentic Henan Wenxian iron stick yam (Jiaozuo, China). Local farmers harvest the Chinese yam each November, and we used samples of Chinese yam freshly dug in November of that year. In this study, dried ginger was selected to explore the mechanism by pungent herbs damage the spleen. Sijunzi Decoction (SJZD), a classic traditional Chinese medicine prescription, is renowned for its spleen-strengthening and qi-replenishing properties, making it a common remedy for spleen deficiency. It consists of *Radix codonopsis* (Dangshen), *Atractylodis macrocephalae rhizoma* (Baizhu), *Poria* (Fuling), and *Glycyrrhizae radix* (Gancao) [15]. Since SJZD has been clinically used to improve symptoms of spleen deficiency [16,17], it was selected as the positive control in this study. Dried ginger was purchased from Zhang Zhongjing Pharmacy (Zhengzhou, China). SJZD was purchased from a local Chinese medicine clinic (Bencao Chinese Medicine Clinic) (Zhengzhou, China). Enzyme-linked immunosorbent assay (ELISA) kits including tumor necrosis factor α (TNF-α) (RX2D310636), interleukin 6 (IL-6) (RX2D302196), interleukin 12 (IL-12) (RX2D301966), interferon-gamma (IFN-γ) (RX302900R), gastrin (GAS) (RX302271R), motilin (MTL) (JRX308376), vasoactive intestinal peptide (VIP), pepsin (RXWB0233-48), and lipase (RXWB0275-96) were purchased from Ruixin Biotechnology Co. (Quanzhou, China). Tissue fixative was purchased from Jiangsu Shitai Experimental Equipment Co., Ltd. (Nanjing, China). D-xylose and D-xylose kit were purchased from Tianjin Huasheng Chemical Reagent Co., Ltd. (Tianjin, China) and Wuhan Eliot elabscience biotechnology Co. (Wuhan, China), respectively. The experimental weight-bearing tin strips were purchased from Beijing Jingdong Century Information Technology (Beijing, China). All remaining reagents were of analytical grade.

### 2.2. Sample Preparation

The beef was cut into evenly sized cubes (average weight 20.0 ± 5.0 g; size 2 × 2 × 2 cm^3^) after removing the external connective tissue and fat. The solid–liquid ratio was 1:3, and the strong fire was transferred to gentle fire for 75 min. The beef was dissolved in the soup by breaking with a tissue grinder (JXFSTPRP-CL, Yueyang, China). Yam soup and beef soup were obtained by the same method. Dry ginger soup was boiled according to the solid–liquid ratio of 1:25. SJZD was purchased from the local Chinese medicine hall, according to the Dangshen, Baizhu, Fuling, and Gancao (2:1.5:1.5:1), soaked for 1 h, simmered for 1 h, and repeated to obtain the SJZD.

### 2.3. Animal Experiments

A total of 36 six-week-old male Sprague-Dawley rats (220 ± 20 g) were purchased from Spectrum Biotechnology Co., Ltd. (Shanghai, China). (Certificate of Conformity SCXK 2024-0001; License No. SCXK 2021-0043). The rats were housed at 23 °C with 40–70% relative humidity and were given food and water normally. All experimental procedures were conducted in accordance with the guidelines of the Institutional Animal Ethics Committee (IAEC), and the experimental protocols received approval from the Experimental Animal Ethics Committee of Research Select Biotechnology (Hangzhou, China) Co., Ltd. (YXSW2410187513).

After the acclimation period, rats were randomly divided into control group (*n* = 6) and model group (*n* = 30). The control group rats were fed a normal standard diet and drank water freely every day. According to the literature Ref. Chen et al. (2018) [18], the model rats were placed in a constant temperature water tank (23 ± 1 °C, water depth of 40 cm, weighted with 10% wire) for exhaustion swimming (exhaustion was defined as sinking at the end of the swimming and not being able to return to the water surface after 10 s). Dietary restriction was imposed (1 day of feeding, 2 days of fasting, with a 3-day cycle) for 14 days. On day 15, after 12 h of fasting, rats in each group were given 5% D-xylose solution (1 mL/100 g), and blood was collected for D-xylose content after 5 h. After successful modeling, a 15-day treatment phase begins. The model rats were randomly divided into groups for gavage administration as follows: the spleen deficiency model group (*n* = 6) was gavaged with distilled water; the beef group (*n* = 6) received soup and beef; the Chinese yam group (*n* = 6) was administered soup and yam; and the SJZD positive control group (SJZD, *n* = 6) and the dried ginger group (*n* = 6) were gavaged with SJZD and dried ginger soup, respectively. Based on human dietary intake (400 g/60 kg/d), the equivalent rat dose was derived using the body surface area conversion factor (6.3) and then concentrated at a 1:5 mass ratio into a gavage solution. The final gavage dose was 0.5 mL/100 g·d^−1^.

### 2.4. Verify Modeling Success

#### 2.4.1. D-Xylose Absorption Experiment

On the 15th day of modeling, all rats were fasted for 12 h. After rats were given 5% D-xylose solution (1 mL/100 g) orally for 5 h, blood was collected from the infraorbital vein and centrifuged. A total of 50 μL of serum, D-xylose solution (50 μL/mL), and distilled water were mixed with 5 mL of cresol violet colorant and placed in boiling water for 5 min. The absorbance of the mixture at 554 nm was measured by UV spectrophotometer using distilled water as a blank control, and the content of D-xylose in rat serum was calculated.

#### 2.4.2. Body Weight and Food Intake

The weight of the rats was measured every three days to monitor the changes in body weight. In a 3-day cycle, there was 1 day of full feeding and 2 days of fasting. On the day of feeding, the amount of food given and the amount remaining when food was withdrawn were weighed, and the difference was calculated. Two rats were housed per cage, and the food was weighed on a cage-by-cage basis.

#### 2.4.3. Health Scores

The general condition of the rats was assessed twice a week in accordance with the criteria presented in Table 1.

### 2.5. Feces Sample Collection

Fresh fecal samples were collected from rats within the 2 h immediately before sacrifice. To collect fresh fecal samples from rats, the skin around the anus was first cleaned with sterile saline to remove bacteria from the environment. Fresh feces were then collected using sterile centrifuge tubes (Thermo Fisher Scientific Inc. Waltham, Massachusetts, United States) autoclaved at 121 °C for 30 min. The entire sampling process was performed on an ultra-clean bench to prevent airborne microbial contamination. Immediately after collection, the samples were rapidly frozen in liquid nitrogen and subsequently stored at −80 °C for 16S rRNA sequencing of the intestinal flora.

### 2.6. Preparation of Serum Samples and Index Measurement

After the last administration of the experimental treatments, the rats fasted overnight and were anesthetized and whole blood and anticoagulated blood were collected from each rat. Whole blood was allowed to stand for 30 min at 25 °C and then centrifuged at 4000 rpm/min for 10 min to obtain serum for subsequent testing [21]. Pancreatic fundus and stomach tissues were frozen at −80 °C for pepsin and pancreatic lipase assays. The spleen index and thymus index were used to illustrate the effect on spleen and thymus weights, calculated as follows:

spleen index (mg/g) = spleen weight (mg)/body weight (g) × 100%

thymus index (mg/g) = thymus weight (mg)/body weight (g) × 100%

### 2.7. Enzyme-Linked Immunosorbent Assay (ELISA)

Enzyme-linked immunosorbent assay (ELISA) was used to detect TNF-α, IFN-γ, IL-6, and IL-12 levels in serum and tissues. Serum biomarkers such as GAS, MTL, VIP, pepsin, pancreatic lipase, and other serum biomarkers were also determined in rat serum. All experiments were performed in strict adherence to the ELISA kit instructions and absorbance values were measured to calculate sample concentrations.

### 2.8. Hematoxylin-Eosin (HE) Staining

Slices of spleen, stomach, small intestine, and colon tissue were prepared and embedded in paraffin. After baking the slices, they were subjected to graded dewaxing, followed by staining with hematoxylin and eosin, respectively, for subsequent histopathological analysis.

### 2.9. 16S rRNA Sequencing

In order to determine the changes in the composition and abundance of intestinal microorganisms, 16S rRNA sequencing was performed on the intestinal contents of rats in this study. The total DNA of the microorganisms was extracted first and the DNA was quantified by Nanodrop; the quality of DNA extraction was detected by 1.2% agarose gel electrophoresis. Then, PCR amplification of target fragments was carried out, using the target sequence of microbial ribosomal RNA as the target, and the corresponding primers were designed according to the conserved regions in the sequence. Sample-specific Barcode sequences were added for PCR amplification of variable regions (single or consecutive multiple) of rRNA genes or specific gene fragments. The amplified products were recovered by magnetic bead purification and then the products were quantified by fluorescence. Sequencing libraries were prepared using Illumina’s TruSeq Nano DNA LT Library Prep Kit ( Illumina, Inc., San Diego, CA, USA) and then subjected to high-throughput sequencing on the machine. To ensure the quality of sequencing, the optimal sequencing length of the target fragments was 200~450 bp.

### 2.10. Data Processing

GraphPad Prism (version 10.1.2, GraphPad Software, Boston, MA, USA) software was used for graphical representation. Experimental data were statistically analyzed using SPSS software (version 25.0, IBM, Almonk, NY, USA). Results were reported as mean ± standard deviation (SD). One-way analysis of variance (ANOVA) was used for multiple comparisons, and comparisons between the two groups were made by Student *t*-test. The *p* < 0.05 was considered as statistical difference.

## 3. Results and Discussion

### 3.1. Evaluation of Spleen-Deficiency Rats

**Figure 1:**
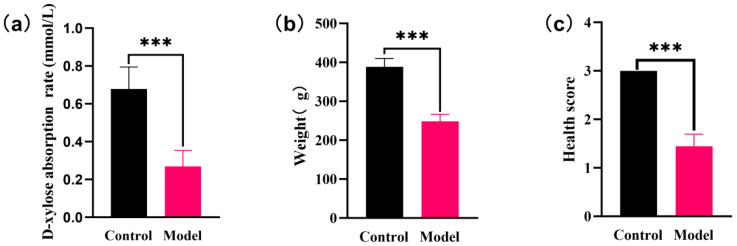
Verifying the effect of the two-factor approach to modeling spleen deficiency. (**a**) Comparison of absorption rate of D-xylose between control group and model group. (**b**) Comparison of body weight between control group and model group. (**c**) Comparison of health scores between control group and model group. All data are expressed as mean ± standard deviation. ∗∗∗ *p* < 0.001.

Currently, D-xylose excretion rate or serum D-xylose absorption rate is used as the main index to evaluate the success of the model [22] (2017 version). After 14 days of modeling, serum D-xylose absorption rate was chosen to be tested on rats in order to exclude the effect of renal function. Compared with the control group, the D-xylose absorption rate of rats in the modeling group was significantly lower than that of the control group (*p* < 0.001) (Figure 1a). The weight of the rats in the model group was significantly lower than those in the control group (*p* < 0.001) (Figure 1b). During the modeling period, control group rats exhibited a robust mental state, white and thick fur, flexible mobility, and regular stool shape. In contrast, SDS rats displayed loose stools, weight loss, reduced activity, diminished appetite, listlessness, clustering behavior, and other such signs. These manifestations in SDS rats were analogous to the symptoms of human spleen deficiency syndrome [23]. According to the health scores of the rats in Table 1, the rats in the model group showed symptoms such as yellow hair, shedding, scanty stools, reduced activity, squinting, and arching of the back (Figure 1c). Therefore, it can be concluded that the spleen deficiency model was successfully established.

### 3.2. Effects of Beef, Yam, and Dried Ginger on Weight, Food Intake, and Health Score in Spleen-Deficiency Rats

Gastrointestinal function should be considered in relation to digestive function, and this is also reflected in weight and food intake. The present study demonstrated that the Chinese herbal formula SJZD effectively ameliorated key symptoms of SDS in rats, including weight loss, reduced food intake, and diminished activity. The coordinated improvement in food intake, body weight, and fecal quality observed in the SJZD and beef groups suggests a potential normalization of the gut–brain axis. This could involve the modulation of appetite-regulating hormones, such as ghrelin and leptin, or serotonergic signaling from the gut, which is known to influence mood and activity levels. Furthermore, the superior integrative recovery observed with SJZD, characterized by steady weight rebound alongside a less-pronounced hyperphagic response compared to the beef group, hints at underlying mechanisms that extend beyond mere caloric intake.

The health scores of the rats improved in each group, with the exception of the dried ginger and model groups. A comprehensive analysis of basic health indicators revealed that the beef group exhibited effects comparable to those of the SJZD group, both effectively enhancing food intake and body weight in spleen deficiency rats. The efficacy of yam was the next best, consistent with traditional Chinese medicine theory, which posits that “sweet flavor herbs strengthen the spleen and benefit the stomach” [24]. In contrast, the pungent flavor of dried ginger did not demonstrate a significant effect on strengthening the spleen; rather, it exhibited a certain inhibitory effect (Appendix A).

### 3.3. Effects of Beef, Yam, and Dried Ginger on Immune Organ Indices and Gastrointestinal Hormones

**Figure 2:**
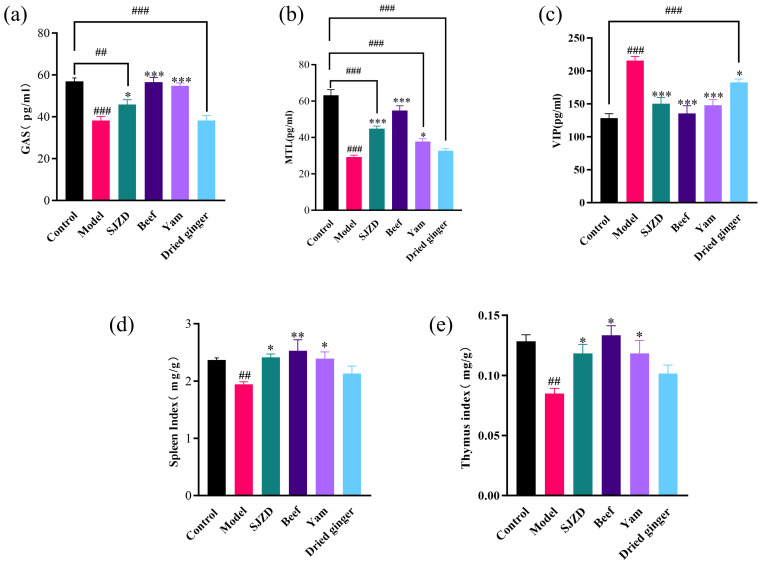
Influence on gastrointestinal hormones and immune organ indices in spleen deficiency rats. (**a**) The changes in gastrin in each group. (**b**) The changes in motilin in each group. (**c**) The changes in vasoactive intestinal peptide in each group. (**d**) The changes in spleen index in each group. (**e**) The changes in thymus index in each group. In comparison to the control group, ## *p* < 0.01, and ### *p* < 0.001. In contrast to the model group, ∗ *p* < 0.05, ∗∗ *p* < 0.01, and ∗∗∗ *p* < 0.001.

Gastrointestinal regulatory peptides are broadly classified into the following two categories: stimulatory peptides, such as gastrin (GAS) and motilin (MTL), and inhibitory peptides, including vasoactive intestinal peptide (VIP) [25]. In the present study, rats with spleen deficiency syndrome (SDS) exhibited a characteristic dysregulation of this peptide axis (Figure 2). Specifically, serum levels of the stimulatory peptides GAS and MTL were significantly reduced compared to the healthy control group (*p* < 0.05), while the level of the inhibitory peptide VIP was markedly elevated (*p* < 0.001). This distinct hormonal profile provides a plausible mechanistic basis for the observed digestive impairments. Gastric acid secretion is stimulated by GAS, which also promotes antral contractions and supports the growth of the gastrointestinal mucosa [26]. MTL modulates the transport of water and electrolytes, enhances gastrointestinal motility, and stimulates the secretion of gastric acid, pancreatic juices, and bile [27,28]. In cases of spleen deficiency (SDD), the levels of GAS and MTL are often disordered [29]. VIP is primarily expressed in the myenteric plexus, mucosal, and submucosal layers of the intestine, where it binds to receptors on intestinal smooth muscle to induce relaxation [30]. Additionally, VIP plays a role in modulating intestinal immune responses [31]. As shown in Figure 2c, VIP exhibited a significantly higher expression level in the model group (*p* < 0.001), which may be associated with gastrointestinal dysfunction in the spleen deficiency state [32]. Both the beef and SJZD interventions effectively normalized this peptide imbalance, restoring GAS, MTL, and VIP to levels comparable to the control group. However, the patterns suggest different primary mechanisms. The beef group’s robust recovery of peptide levels, coinciding with its significant increase in food intake, implies a nutrient-driven, feedback-dependent mechanism, where increased caloric and protein substrate may stimulate enteroendocrine cell secretion via neurohormonal reflexes. In contrast, the SJZD group achieved steady weight rebound and hormonal normalization without a concomitant hyperphagic response. This points to a more direct, multi-target regulatory action of the herbal formula, potentially modulating enteroendocrine cell activity, vagal afferent signaling, or central appetite circuits to proactively re-establish gut–brain axis homeostasis. A decrease in the spleen index and thymus index may indicate atrophy of the immune organs [33]. This suggests that both immune function and gastrointestinal hormone secretion are impaired in the model group (Figure 2). Following the intervention treatment, all indices of the SJZD, beef group, and yam group showed significant rebounds (*p* < 0.05) and approached normal levels. This indicates that these groups exhibited notable improvements in the immune and gastrointestinal functions of spleen deficiency rats.

### 3.4. Effects of Beef, Yam, and Dried Ginger on Immune Indices in Spleen-Deficiency Rats

**Figure 3:**
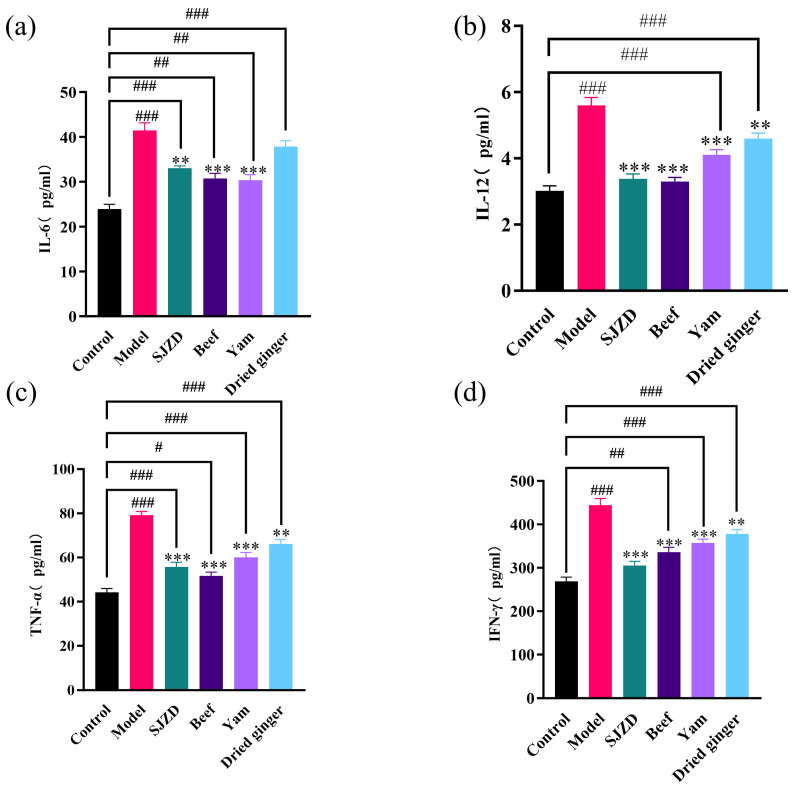
Influence on inflammatory cytokines in spleen deficiency rats. (**a**) The changes in IL-6 in each group. (**b**) The changes in IL-12 in each group. (**c**) The changes in TNF-α in each group. (**d**) The changes in IFN-γ in each group. In comparison to the control group, # *p* < 0.05, ## *p* < 0.01, and ### *p* < 0.001. In contrast to the model group, ∗∗ *p* < 0.01, and ∗∗∗ *p* < 0.001.

The proposed pathway linking spleen deficiency to gastrointestinal dysfunction involves a cascade of immune events. First, T helper 1/T helper 2 (Th1/Th2) homeostasis, which is crucial for maintaining immune balance, becomes dysregulated in spleen deficiency syndrome (SDS). This dysregulation results in the abnormal elevation of pro-inflammatory factors [34,35]. Subsequently, SDS activates splenic macrophages. These activated macrophages typically secrete TNF-α, which in turn induces the secretion of IL-6, thereby initiating inflammation [36,37]. As illustrated in Figure 3, this cascade leads to significantly higher serum levels of IL-6, IL-12, TNF-α, and IFN-γ in the SDS model group compared to the control group (*p* < 0.05). Ultimately, this persistent inflammatory milieu may directly suppress gastrointestinal motility and enteroendocrine function, while also promoting immune cell infiltration and potential tissue damage in the gut and immune organs. Following intervention treatment, the levels of the aforementioned inflammatory factors were significantly reduced (*p* < 0.001). Notably, the trends in the changes in these four immune factors were similar, with SJZD restoring IL-12 and IFN-γ to normal levels, followed closely by the beef group. This indicates that immune suppression was enhanced in spleen deficiency rats, potentially through signaling pathways that regulate Th1/Th2 immune balance [38]. Although dried ginger also exhibited a certain degree of improvement in the immune system of spleen deficiency rats, the effect was limited, and a significant difference remained compared to the control group (*p* < 0.001).

### 3.5. Effects of Beef, Yam, and Dried Ginger on Proteins and Related Enzymes

**Figure 4:**
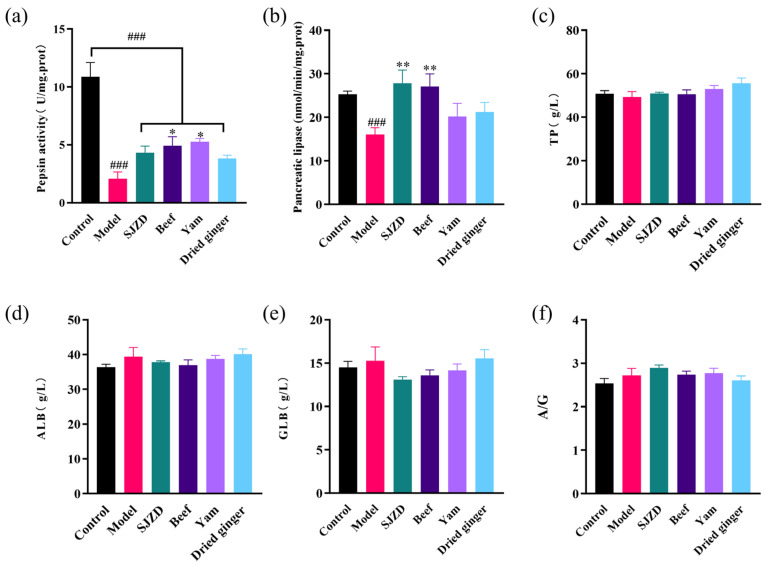
Influence on proteins and related enzymes in spleen-deficiency rats. (**a**) Pepsin activity. (**b**) Pancreatic lipase. (**c**) The changes in TP in each group. (**d**) The changes in ALB in each group. (**e**) The changes in GLB in each group. (**f**) The changes of A/G in each group. In comparison to the control group, ### *p* < 0.001. In contrast to the model group, * *p* < 0.05 and ** *p* < 0.01.

Figure 4 illustrates that pepsin activity in the SDS group was significantly lower than that in the control group (*p* < 0.001) and pancreatic lipase activity was also lower than that in the control group. These findings suggest that a state of spleen deficiency may lead to digestive dysfunction, subsequently impairing the body’s ability to digest and absorb proteins and fats. While there was a significant increase in pepsin activity in the beef and yam groups (*p* < 0.05), pepsin activity across all treatment groups remained significantly lower than that of the control group (*p* < 0.001). This phenomenon may reflect a protective mechanism of the body, as excessive pepsin secretion during gastric injury could be detrimental to the stomach itself. Furthermore, following intervention treatment, pancreatic lipase activity was significantly elevated (*p* < 0.01) in both SJZD and beef groups, compared to the spleen deficiency group (Figure 4b). No significant differences (*p* > 0.05) were observed among the groups regarding total protein (TP), albumin (ALB), globulin (GLB) levels, and the albumin to globulin ratio (A/G), which reflect protein metabolism. This lack of significant variation may be attributed to either the short duration of the experimental period or the low specificity of these indicators for spleen deficiency. The reduction in pepsin and pancreatic lipase likely reflects impaired exocrine function, potentially due to suppressed synthesis or secretion from gastric chief cells and pancreatic acinar cells, respectively. The significant increase in enzyme activity following interventions with SJZD, beef, and yam suggests these substances may contain bioactive components that stimulate digestive gland secretion, enhance enzymatic activation, or improve the metabolic milieu of these cells.

### 3.6. Effects of Beef, Yam, and Dried Ginger on Intestinal Flora

**Figure 5:**
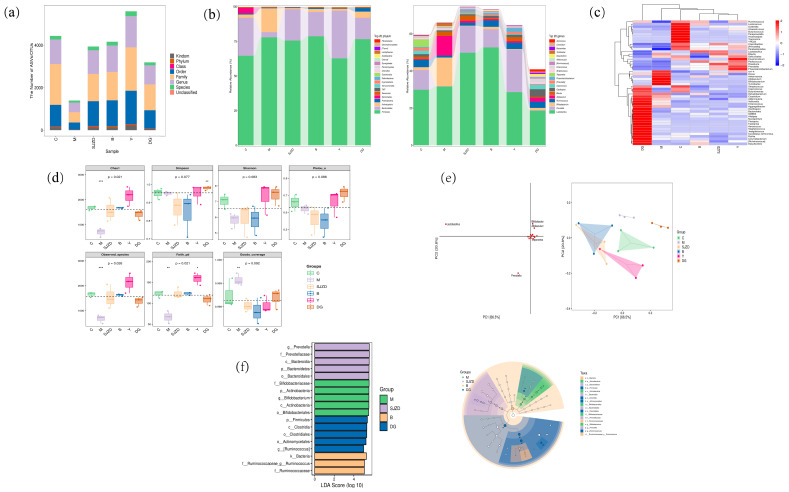
Influence on intestinal flora in spleen deficiency rats. (**a**) Plot of taxonomic unit number of intestinal flora. (**b**) Composition of intestinal flora at the phylum and genus level in different groups of rats. (**c**) The heatmap of species on genus level. (**d**) Alpha diversity analysis of the composition of rat intestinal flora. * *p* < 0.05, ** *p* < 0.01 and *** *p* < 0.001. (**e**) Beta diversity analysis of rat intestinal flora composition PCA. (**f**) The results of LEfSe data analysis of rat intestinal flora in each group. The left panel is histogram of LDA value distribution, and the right panel is evolutionary branching of LEfSe analysis. C, M, B, Y, and DG represent the control, model, beef, yam, and dried ginger groups, respectively.

In recent years, numerous studies have reported that gut microbiota plays an increasingly significant role in SDS, regulating gastrointestinal functions such as absorption, metabolism, intestinal barrier integrity, and neuroendocrine functions [39]. As illustrated in Figure 5a, the diversity of gut microbiota in the model group was significantly reduced (*p* < 0.05), indicating a loss of microbial richness and evenness associated with the diseased state. However, following yam intervention, the diversity of the microbiota was significantly increased, suggesting a restorative effect on microbial community resilience (*p* < 0.05). The composition of the intestinal microbiota is predominantly characterized by the phyla Firmicutes and Bacteroidetes [40,41]. From the distribution of these phyla in Figure 5b, it is evident that, compared to the control group, the relative abundance of Bacteroidetes in the model group exhibited a downward trend, resulting in a reduced ratio of Bacteroidetes-to-Firmicutes (B/F). Studies have indicated that the Bacteroidetes-to-Firmicutes ratio in gut microbiota is associated with inflammation and is believed to contribute to conditions such as diarrheal irritable bowel syndrome [42,43,44]. Compared to the model group, the relative abundance of Bacteroidetes was significantly increased in each treatment group (*p* < 0.05), with the B/F ratio in the SJZD group being closest to that of the control group. The relative abundance of the genus Aeromonas was significantly elevated in the model group. This proliferation could directly exacerbate intestinal dysfunction through potential virulence factors or by disrupting commensal niches. Notably, we observed a significant increase in the relative abundance of Bifidobacterium in the SDS group, which may represent a compensatory response to systemic inflammation. This finding aligns with the observations reported by Dong (2024) [45]. To elucidate the shifts in taxonomic composition across treatment groups, we conducted a heatmap analysis of the top 30 most abundant species. The SJZD group demonstrated the greatest similarity to the control group, indicating its efficacy in restoring the composition of intestinal microbiota and alleviating symptoms of spleen deficiency in rats, thereby approaching normal physiological conditions. Conversely, the dry ginger group exhibited a high degree of similarity to the model group (Figure 5c).

The Alpha index serves as a metric for assessing the richness and diversity of microbial communities (Figure 5d). In our study, we employed the Chao1 index and the observed species index to evaluate species richness, while the Shannon and Simpson indices were utilized to gauge species diversity. Additionally, Faith’s PD index was applied to assess evolutionary-based species diversity. The Pielou_e index and Goods_coverage provide insights into species distribution uniformity and the extent to which sequencing results represent the true microbial community, respectively. Our findings reveal that the model group exhibited significantly lower alpha diversity compared to the control group (*p* < 0.001). Principal component analysis (PCA) of the intestinal microbiota indicates a substantial divergence in microbiota structure between the model and control groups, with yam treatment facilitating a shift in the microbiota structure closer to that of the control group. The left diagram illustrates a notable concentration of the genus Prevotella in the yam treatment (Figure 5e). LeFSe analysis (LDA score > 4) was employed to identify key differential bacterial groups across various treatment groups [46]. As illustrated in Figure 5f, the SJZD group was predominantly characterized by g_Prevotella, f_Prevotellaceae, c_Bacteroidia, *p*_Bacteroidetes, and o_Bacteroidales. In contrast, the model group displayed distinct microbial traits, primarily dominated by f_Bifidobacteriaceae, *p*_Actinobacteria, g_Bifidobacterium, c_Actinobacteria, and o_Bifidobacteriales. Notably, the beef group exhibited a unique composition, including k_Bacteria, f_Ruminococcaceae_g_Ruminococcus, and f_Ruminococcaceae.

### 3.7. Effects of Beef, Yam, and Dried Ginger on the Spleen, Stomach, Small Intestine, and Colon

**Figure 6:**
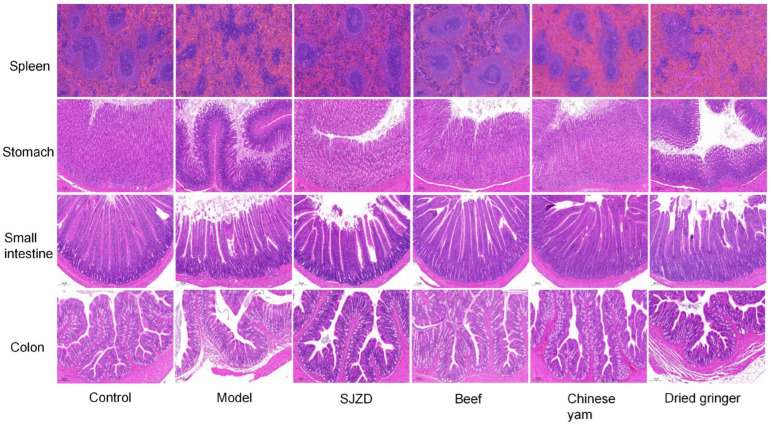
Morphological observation of the spleen, stomach, small intestine, and colon in various groups of rats by HE staining (scale bar 200 μm).

Furthermore, we studied the effects of beef, yam, and dried ginger on rats with spleen deficiency from the perspective of histopathological changes. HE staining revealed significant damage to the spleen, stomach, small intestine, and colon tissues in the model group rats (Figure 6). In contrast, the control group exhibited a clear spleen structure, with distinct boundaries between the red pulp and white pulp, which were neatly organized. Conversely, in the model group, the boundaries between the red pulp and white pulp were indistinct, characterized by irregular arrangement and shape, disrupted white pulp structure, and abnormal morphology of splenic corpuscles. All treatment groups, except the dry ginger group, demonstrated varying degrees of recovery, with the beef group achieving the most favorable outcomes. HE staining of gastric tissue indicated that the gastric wall in the control group was smooth, featuring neatly arranged glands. Although no organic damage was observed in the model group compared to the control group, the gastric mucosal surface exhibited severe damage, with irregular gland arrangement. Both the dry ginger and model groups displayed infiltration of inflammatory cells. In the control group, the small intestinal villi were abundant and densely packed, with intact structures and well-developed glands. However, in the model group, the intestinal villi appeared disordered and stacked, with thinner intestinal walls and increased viscosity of the luminal contents. Compared to the model group, all treatment groups exhibited a greater abundance of small intestinal villi and reduced viscosity of the luminal substances, indicating significant improvement. The control group presented clear colonic crypts, a well-organized colonic epithelial cell structure, and no mucosal damage. In contrast, the model group showed edema of the lamina propria in the colonic villi, reduced cell numbers, disorganized cell arrangement, and separation of the lamina propria from the mucosal epithelium. Additionally, submucosal edema and cellular infiltration were noted. All treatment groups, with the exception of the dry ginger group, exhibited significant alleviation of these pathological changes.

## 4. Conclusions

This study systematically investigated the effects of beef, yam, and dried ginger on rats with SDS. Our research confirmed that beef and yam significantly reversed the pathological state of SDS, with efficacy comparable to that of the traditional spleen-tonifying formula SJZD. From a mechanistic perspective, beef and yam enhance the gastrointestinal function by increasing GAS and MTL levels and enhancing pepsin and lipase activity. At the same time, they regulate immune homeostasis by reducing pro-inflammatory cytokine levels (IL-6, IL-12, TNF-α, and IFN-γ) and restoring the spleen index and thymus index, which mitigates systemic low-grade inflammation. Additionally, they correct intestinal dysbiosis by increasing the abundance of Bacteroidetes, optimizing the Bacteroidetes-to-Firmicutes (B/F) ratio, and enriching beneficial bacterial communities such as *Prevotella*, thereby promoting a healthier gut microbiota structure. In sharp contrast, pungent dried ginger failed to alleviate the SDS pathological process; no significant improvements were observed in metabolic indicators, immune parameters, or digestive function. In conclusion, beef and Chinese yam, as medicinal and food ingredients, can significantly improve the symptoms of spleen deficiency through multiple pathways, and their mechanisms of action include enhancement of digestive function, immunomodulation, and restoration of intestinal microecology. This provides guidance for the subsequent development of health products by combining different medicinal and food ingredients.

## Figures and Tables

**Table 1:** Health scoring criteria.

Score	Fecal Pattern	Mental Status	Skin	Tail
1	Loose	Curled up, clumped	Shedding yellowing	Paler
2	Soft	Arched back, squinting	Fluffy and rough	Pale
3	Firm	Lively	Smooth and silky	Reddish

Note: the references cited in this review form have undergone modifications [19,20].

## Data Availability

The original contributions presented in the study are included in the article, further inquiries can be directed to the corresponding author.

## Appendix A

Effects of beef, yam, and dried ginger on spleen-deficiency rats.

**Table:**

Index	Time	Control	Model	SJZD	Beef	Yam	Dried Ginger
Food intake	MD1	28.35 ± 1.21 ^a^	27.53 ± 0.76 ^a^	27.17 ± 3.03 ^a^	28.63 ± 4.02 ^a^	24.02 ± 2.52 ^a^	28.60 ± 1.10 ^a^
	MD4	29.01 ± 2.15 ^a^	35.18 ± 1.68 ^a^	34.85 ± 1.13 ^a^	35.17 ± 1.73 ^a^	34.62 ± 1.71 ^a^	34.55 ± 2.54 ^a^
	MD7	29.88 ± 1.03 ^c^	33.73 ± 1.60 ^ab^	33.05 ± 1.87 ^ab^	34.23 ± 0.89 ^a^	31.42 ± 1.15 ^bc^	34.80 ± 1.75 ^a^
	MD10	30.98 ± 1.30 ^b^	35.53 ± 1.75 ^a^	35.22 ± 1.30 ^a^	36.63 ± 1.11 ^a^	33.93 ± 1.92 ^a^	34.33 ± 1.73 ^a^
	MD13	31.10 ± 1.46 ^b^	35.45 ± 2.05 ^a^	33.22 ± 1.73 ^ab^	33.70 ± 1.62 ^ab^	31.87 ± 3.19 ^ab^	33.45 ± 2.50 ^ab^
	TD3	33.15 ± 3.36 ^b^	35.00 ± 3.00 ^ab^	36.52 ± 5.82 ^ab^	40.62 ± 2.37 ^a^	32.35 ± 2.78 ^b^	31.25 ± 3.24 ^b^
	TD7	33.90 ± 3.06 ^bc^	32.17 ± 4.40 ^bc^	37.55 ± 3.32 ^b^	44.88 ± 3.99 ^a^	34.48 ± 2.19 ^bc^	29.50 ± 2.50 ^c^
	TD10	35.53 ± 3.43 ^bc^	31.80 ± 2.80 ^c^	38.37 ± 0.98 ^b^	47.48 ± 1.51 ^a^	36.20 ± 2.93 ^b^	26.27 ± 2.23 ^d^
	TD13	36.25 ± 2.73 ^b^	30.07 ± 4.90 ^c^	38.43 ± 4.47 ^b^	48.95 ± 1.75 ^a^	36.67 ± 2.21 ^b^	23.40 ± 4.86 ^d^
Weight	MD0	283.12 ± 9.03 ^a^	272.63 ± 5.45 ^a^	272.77 ± 13.45 ^a^	270.92 ± 12.57 ^a^	273.67 ± 8.69 ^a^	276.00 ± 9.40 ^a^
	MD3	301.93 ± 12.97 ^a^	256.17 ± 7.92 ^b^	259.92 ± 9.41 ^b^	260.23 ± 12.10 ^b^	258.03 ± 8.15 ^b^	266.73 ± 9.00 ^b^
	MD6	333.05 ± 13.78 ^a^	234.88 ± 12.48 ^b^	231.73 ± 12.14 ^b^	234.62 ± 9.97 ^b^	234.30 ± 4.23 ^b^	244.22 ± 9.33 ^b^
	MD9	351.55 ± 16.90 ^a^	220.78 ± 10.88 ^b^	222.47 ± 10.43 ^b^	221.12 ± 11.47 ^b^	224.90 ± 11.73 ^b^	231.88 ± 11.63 ^b^
	MD12	373.77 ± 19.73 ^a^	214.63 ± 14.30 ^b^	214.78 ± 11.30 ^b^	216.93 ± 12.51 ^b^	216.72 ± 14.38 ^b^	222.85 ± 13.40 ^b^
	TD2	388.72 ± 21.60 ^a^	238.13 ± 9.80 ^b^	254.18 ± 14.20 ^b^	256.95 ± 22.00 ^b^	245.47 ± 20.59 ^b^	254.92 ± 18.34 ^b^
	TD5	384.25 ± 43.11 ^a^	223.95 ± 7.46 ^b^	228.52 ± 23.60 ^b^	244.27 ± 16.96 ^b^	228.73 ± 18.03 ^b^	251.48 ± 10.50 ^b^
	TD8	409.15 ± 30.21 ^a^	232.08 ± 14.16 ^c^	253.38 ± 22.48 ^b^	278.32 ± 24.99 ^b^	260.27 ± 23.81 ^b^	255.77 ± 26.56 ^bc^
	TD11	432.98 ± 26.74 ^a^	257.42 ± 19.29 ^cd^	297.40 ± 10.91 ^b^	286.87 ± 22.28 ^bc^	267.35 ± 16.27 ^cd^	245.10 ± 38.30 ^d^
	TD14	453.37 ± 31.80 ^a^	274.23 ± 15.31 ^d^	353.30 ± 20.50 ^b^	322.80 ± 25.63 ^bc^	310.75 ± 18.19 ^c^	248.25 ± 32.37 ^d^
Health score	TD0	3.00 ± 0.00 ^a^	1.29 ± 0.29 ^b^	1.33 ± 0.26 ^b^	1.42 ± 0.13 ^b^	1.50 ± 0.16 ^b^	1.46 ± 0.19 ^b^
	TD2	3.00 ± 0.00 ^a^	1.50 ± 0.16 ^c^	1.50 ± 0.22 ^c^	1.58 ± 0.13 ^bc^	1.75 ± 0.16 ^b^	1.54 ± 0.19 ^bc^
	TD5	3.00 ± 0.00 ^a^	1.71 ± 0.19 ^c^	1.67 ± 0.13 ^c^	1.96 ± 0.19 ^b^	1.75 ± 0.16 ^bc^	1.67 ± 0.13 ^c^
	TD8	3.00 ± 0.00 ^a^	1.67 ± 0.30 ^d^	1.96 ± 0.19 ^bc^	2.17 ± 0.13 ^b^	2.00 ± 0.16 ^bc^	1.88 ± 0.31 ^cd^
	TD11	3.00 ± 0.00 ^a^	1.96 ± 0.10 ^c^	2.38 ± 0.14 ^b^	2.42 ± 0.13 ^b^	2.29 ± 0.19 ^b^	1.92 ± 0.30 ^c^
	TD14	3.00 ± 0.00 ^a^	1.92 ± 0.20 ^d^	2.08 ± 0.20 ^bcd^	2.29 ± 0.10 ^bc^	2.38 ± 0.34 ^b^	2.04 ± 0.19 ^cd^

Note: MD and TD represent the specific number of days during the modeling period and treatment period, respectively. For each row different lowercase letters in the table indicate significant differences between samples (*p* < 0.05).
